# Identification of Site-Specific Adaptations Conferring Increased Neural Cell Tropism during Human Enterovirus 71 Infection

**DOI:** 10.1371/journal.ppat.1002826

**Published:** 2012-07-26

**Authors:** Samuel Cordey, Tom J. Petty, Manuel Schibler, Yannick Martinez, Daniel Gerlach, Sandra van Belle, Lara Turin, Evgeny Zdobnov, Laurent Kaiser, Caroline Tapparel

**Affiliations:** 1 Laboratory of Virology, Division of Infectious Diseases and Division of Laboratory Medicine, University Hospitals of Geneva, Geneva, Switzerland; 2 Department of Medicine, University of Geneva Medical School, Geneva, Switzerland; 3 Department of Genetic Medicine and Development, University of Geneva Medical School, Geneva, Switzerland; 4 Swiss Institute of Bioinformatics, Geneva, Switzerland; 5 Department of Pathology and Immunology, Faculty of Medicine, University of Geneva, Geneva, Switzerland; 6 Research Institute of Molecular Pathology (IMP), Vienna, Austria; 7 Imperial College London, South Kensington Campus, London, United Kingdom; University of California, Irvine, United States of America

## Abstract

Enterovirus 71 (EV71) is one of the most virulent enteroviruses, but the specific molecular features that enhance its ability to disseminate in humans remain unknown. We analyzed the genomic features of EV71 in an immunocompromised host with disseminated disease according to the different sites of infection. Comparison of five full-length genomes sequenced directly from respiratory, gastrointestinal, nervous system, and blood specimens revealed three nucleotide changes that occurred within a five-day period: a non-conservative amino acid change in VP1 located within the BC loop (L97R), a region considered as an immunogenic site and possibly important in poliovirus host adaptation; a conservative amino acid substitution in protein 2B (A38V); and a silent mutation in protein 3D (L175). Infectious clones were constructed using both BrCr (lineage A) and the clinical strain (lineage C) backgrounds containing either one or both non-synonymous mutations. *In vitro* cell tropism and competition assays revealed that the VP1_97_ Leu to Arg substitution within the BC loop conferred a replicative advantage in SH-SY5Y cells of neuroblastoma origin. Interestingly, this mutation was frequently associated *in vitro* with a second non-conservative mutation (E167G or E167A) in the VP1 EF loop in neuroblastoma cells. Comparative models of these EV71 VP1 variants were built to determine how the substitutions might affect VP1 structure and/or interactions with host cells and suggest that, while no significant structural changes were observed, the substitutions may alter interactions with host cell receptors. Taken together, our results show that the VP1 BC loop region of EV71 plays a critical role in cell tropism independent of EV71 lineage and, thus, may have contributed to dissemination and neurotropism in the immunocompromised patient.

## Author Summary

Human enterovirus-71 (EV71) has been the cause of major hand-foot-and-mouth disease outbreaks, particularly in the Asia-Pacific region. EV71 infection can also disseminate to the central nervous system and result in meningo-encephalitis. Despite intensive epidemiological screening, as well as experimentation in animal models, viral factors contributing to neurotropism remain ill-defined. We describe here the analysis of the full-length genomes of EV71 from different infection sites in an immunocompromised host with disseminated disease. Our data highlight a critical amino acid change within the EV71 VP1 protein that could potentially lead to dissemination and neurotropism during natural infections. This hypothesis was confirmed *in vitro* through reverse genetic experiments in different EV71 lineages and by *in silico* modelling. To our knowledge, this study provides the first genome-wide analysis of EV71 evolution and dissemination within a single human host over the course of an infection, and highlights how the emergence of mutations at critical regions of the viral genome can potentially lead to new phenotypes and neurovirulence.

## Introduction

In humans, enteroviruses target a variety of different organs causing gastrointestinal, respiratory, myocardial, and central nervous system (CNS) diseases [1], [2]. The ability of enteroviruses other than poliovirus to cause neurological complications is restricted to a limited number of serotypes that include enterovirus 71 (EV71) [3], [4]. EV71 is of particular interest since it can cause major hand-foot-and-mouth disease outbreaks, such as those recently reported across the Asia-Pacific countries [5]–[8]. Nevertheless, EV71 dissemination to the CNS remains a rare event, as demonstrated by the relatively small proportion of meningo-encephalitis among millions of hand-foot-and-mouth disease cases [9]–[12].

For poliovirus, CNS invasion is thought to occur either through disruption of the blood-brain barrier or via retrograde axonal transport [8]. For EV71, experimental studies in mouse models using adapted strains suggest that the virus has the propensity to invade the CNS through retrograde axonal transport and that hematogenous transport might represent only a minor route of transmission [13]–[15]. However, the observations in mouse models do not necessarily reflect how CNS invasion occurs during human infections.

Neutrotropic enteroviruses need to escape the host defences to reach the CNS. The absence of pre-existing protective immunity, together with a relatively deficient innate immunity, is considered as the first step toward high blood viremia that will then lead to a secondary invasion of the CNS [16]. This explains why young children present more severe diseases. An inefficient immune response could also be the result of a high inoculum size, leading to an overwhelming replication and viremia. However, neurotropism is a multistep event that requires the virus not only to sustain high replication levels, but also to locate a permissive cell type within the CNS. Viral factors contributing to neurotropism have been intensively studied *in vitro* and in animal models *in vivo* using poliovirus or non-polio EVs [15]–[23], but still remain ill-defined. Until now, to the best of our knowledge, EV71 virulence factors and adaptation have not been studied directly from clinical samples during natural human infections and it remains unknown whether secondary seeding from the primary site is only a fortuitous event or if it is associated with specific viral genomic adaptation within the human host.

In this study, we analyzed the genomes of EV71 from different sites of infection in an immunocompromised host with disseminated disease. This provided a unique opportunity to investigate any potential intra-host adaptation following natural human infection and to assess whether enterovirus needs to harbor specific genomic features in order to sustain dissemination. After sequence analysis of the collected specimens, amino acid changes observed in the viral proteins VP1 and 2B and possibly associated with neurotropism were further studied both *in vitro* using a series of different constructs and *in silico* using comparative models of EV71 VP1.

## Results

### Case Description

A 38-year-old man with chronic lymphocytic leukemia and recently treated with four courses of chemotherapy, including rituximab, was hospitalized with fever and respiratory symptoms. Five days before admission, he developed fever (39°C), odynophagia, chills, dyspnoea with wheezing, cough and sputum. The total immoglobulin G level in blood was low at 2.74 g/L (normal range, 6.06–13.18 g/L), as were the IgM (0.1 g/L; normal range, 0.29–3.25) and IgA (0.17 g/L; normal range, 0.66–3.99) levels. Despite intravenous wide-spectrum antibiotic and antifungal treatment, fever persisted together with diarrhea. The appearance of meningeal signs prompted a lumbar puncture that revealed a slight inflammation with six white blood cells/mm^3^, but normal protein and glucose levels. Microbiological investigations revealed a positive enterovirus RT-PCR signal in the lower respiratory specimens (BAL), plasma, cerebrospinal fluid (CSF), and stools. Viral culture was positive for enterovirus in the respiratory tract and stools. Additional extensive microbiological investigations were all negative for any other bacterial, fungal, or viral infections. Disseminated enteroviral disease was diagnosed and the clinical condition improved rapidly after immunoglobulins were infused. This infusion was followed by a clearance of the infection in blood as shown by a negative RT-PCR assay at day 7 after infusion without relapse or evidence of persisting enteroviral infection.

### Genomic Investigations

The full-length enterovirus genomes were sequenced directly from BAL, stool, plasma (at days 0 and 4) and CSF specimens (Genbank accession numbers: EU414331 to EU414335). A whole genome BLAST search and a phylogenetic tree with available EV full-length genomes (http://www.picornaviridae.com/enterovirus/hev-a/hev-a_seqs.htm) revealed that this strain clusters with other EV71 serotypes within the human EV-A species. This serotyping was confirmed by immunofluorescence with an anti-EV71 monoclononal antibody applied on the BAL and the stool isolates grown in Vero cells.

This clinical enterovirus strain is related to the genogroup C1. Its full-length polyprotein sequence was then compared to publicly available full-length EV71 sequences and linked with the identified associated clinical conditions. This large-scale inter-host analysis did not identify any genomic features that could be related to specific clinical features or to disease severity. This finding indirectly supported the completion of an intra-host full-length genome analysis to find critical residues that could promote virus dissemination and invasion of the CNS.

### Site-Specific Genome Analysis

Genomic DNA sequences and polyprotein comparisons of the five different specimens revealed two non-synonymous substitutions at positions 662 and 1050, and one synonymous substitution at position 1906 of the EV71 polyprotein (GenBank accession number: AAB39968.1). These positions correspond to amino acid 97 of VP1, 38 of 2B, and 175 of 3D (Table 1). None of these three mutations had any effect on the RNA secondary structure in the specific regions (data not shown). No other mutations were observed.

**Table 1:** Genome evolution at the nucleotide and amino-acid level according to the time and site of sampling.

Time	Site	VP1 nt*	VP1 aa*	2B nt	2B aa	3D nt	3D aa
		1985 (290)	662 (97)	3149 (113)	1050 (38)	5718 (525)	1906 (175)
Day 0	BAL	T	Leu	T	Val	C	Leu
Day 1	Plasma 1	G	Arg	C	Ala	C	Leu
Day 1	Stool	T + G	Leu + Arg	C	Ala	C + T	Leu
Day 4	Plasma 2	G	Arg	C	Ala	C	Leu
Day 5	CSF	G	Arg	C	Ala	C	Leu

Positions of nucleotide and amino acid substitutions are listed in reference to both the full-length EV71 polyprotein (number on left of column) and within the affected protein (in parenthesis). BAL: bronchoalveolar lavage sample; CSF: cerebrospinal fluid sample; nt: nucleotide position; aa: amino acid position.

*: Positions are indicated relative to EV71 BrCr strain (GenBank accession number: U22521).

Of note, real-time RT-PCR performed on plasma samples collected at days 1, 5 and 7 presented 27, 32 and 45 ct values, respectively, and immunoglobulins were injected at day 5.

#### VP1_97_ leucine to arginine substitution

Compared to the initial sampling (day 0) from the lower respiratory tract, which contained a leucine at position 97 of the VP1 capsid protein (herein referred to as VP1_97L_), an arginine was present at this position (VP1_97R_) in the day 1 plasma and in the CSF sampled at day 5. In both specimens (day 1 plasma and day 5 CSF), a mixed population was not observed and only VP1_97R_ was present. The stool specimen sampled at day 1 contained both residues at this position, suggesting that the stool harbored a mixture of these two different species. In stool, viral culture isolated the VP1_97R_ subspecies as the unique and dominant strain, whereas in the respiratory specimen only the VP1_97L_ subspecies was isolated.

This leucine to arginine substitution (L97R), located within the VP1 capsid protein, is a non-conservative change that replaces a hydrophobic non-polar residue with one that is positively charged. Based on sequence alignments to other picornavirus VP1 proteins and the comparative models of EV71 VP1 that we generated, residue 97 is located in the BC loop, a known dominant immunogenic site [24]–[30] situated near the putative cellular receptor binding site. An extensive alignment of 952 full-length VP1 sequences of EV71 isolates from GenBank, including our clinical specimens, revealed that this L97R substitution has previously been identified in only two other isolates, one from a meningitis case (GenBank accession number: AAB63227) and another from a case with an unspecified condition (GenBank accession number: AAF13503) [31]. Of note, it has been established that the amino acid sequence of the VP1 BC loop (residues 93–104) is an important determinant of poliovirus host adaptation [32] and that residue changes within this antigenic site show an association with mouse neurovirulence [33].

#### 2B_38_ valine to alanine substitution

The second amino acid substitution occurred in protein 2B, known to enhance cell membrane permeability during viral infection. The neutral non-polar valine residue at position 38 (2B_38V_) was replaced by another neutral non-polar alanine residue (2B_38A_), resulting in a conservative substitution (V38A). This 2B_38A_ substitution was present as the dominant species in the consensus sequence from the stool, blood, and CSF samples. Furthermore, multiple sequence alignments of 291 EV71 polyprotein sequences in Genbank revealed that most (285) circulating strains contain an alanine at this position, while the remaining sequences either contain a valine (Genbank accession numbers: ABC69251; ABW98513; ABW98514; ACB56581; ACM47545), or threonine (ABW98520). In the case of our clinical isolates, it seems that EV71 reverted to the common 2B_38A_ sequence.

### Immune Response

To investigate whether these two changes could play a role in immune escape, we established quantified viral stocks in Vero cells with the BAL (*VP1*_97L_-*2B*_38V_) and the stool (*VP1*_97R_-*2B*_38A_) isolates, respectively. Conservation of these two substitutions after cell passage was confirmed by re-sequencing. The two isolates were tested for seroneutralization in the presence of the patient's serum (sampled at day 4) at a time when the *VP1*_97R_ substitution was already present in plasma. Neither the BAL isolate nor the stool isolate were neutralized by the patient's serum (Table 2). A negative complement fixation assay confirmed a poor antibody response against enterovirus (data not shown). Of note, in the presence of the anti-EV71 monoclonal antibody, the growth of the *VP1*_97R_-*2B*_38A_ stool isolate was inhibited at a dilution <1∶30, whereas the BAL isolate remained insensitive, thus arguing against an immune escape advantage resulting from the VP1_97R_ substitution.

**Table 2:** Seroneutralization assay with the patient's serum.

*Clinical isolate*	*Antibody source*	
	EV71mAB inhibitory dilution	Patient serum (d4) inhibitory dilution
Stool	<1∶30	<1∶5**
BAL	<1∶10*	<1∶5**

*: smaller dilution not tested;

**: Patient serum was toxic for cells at a dilution <1∶5.

d4: patient serum sampled at day 4.

BAL: bronchoalveolar lavage.

### Cell Tropism of the BAL and Stool EV71 Isolates

**Figure 1:**
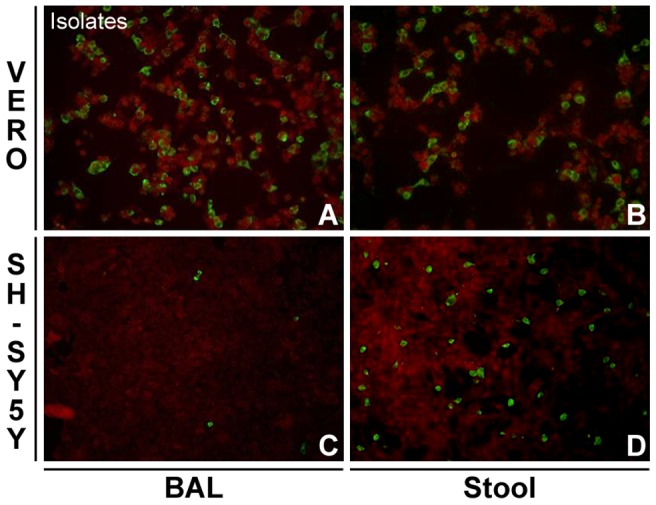
Replication efficiency of the lower respiratory tract (BAL) and stool isolates in Vero versus SH-SY5Y cells. Vero cells (A–B) and SH-SY5Y cells (C–D) were infected with equivalent concentrations of BAL and stool isolates and replication was assessed by immunofluorescence with anti-EV71 monoclonal antibody.

To investigate the potential implication of the mutations on tissue tropism, we then inoculated the *VP1*_97L_-*2B*_38V_ (BAL) and *VP1*_97R_-*2B*_38A_ (stool) isolates on three cell lines (astrocytoma [U-87 MG], neuroepithelioma [SK-N-MC], and neuroblastoma cell lines [SH-SY5Y]) previously used as references to confirm the ability of poliovirus [34], [35] or EV71 [23], [36] to infect cells of neural origin. Figure 1 shows that the stool isolate presents a strong replication advantage over the respiratory tract specimen in cells of neuroblastoma origin, whereas the two isolates replicate in similar fashion in the astrocytoma cell line (data not shown). Of note, neither of the two isolates was able to grow in neuroepithelioma cell lines, although a wild type poliovirus used as control grew easily under the same conditions (data not shown).

### Role of the VP1_97R_ and 2B_38A_ Mutations Investigated by Reverse Genetics in the Clinical Isolate Backgrounds

**Figure 2:**
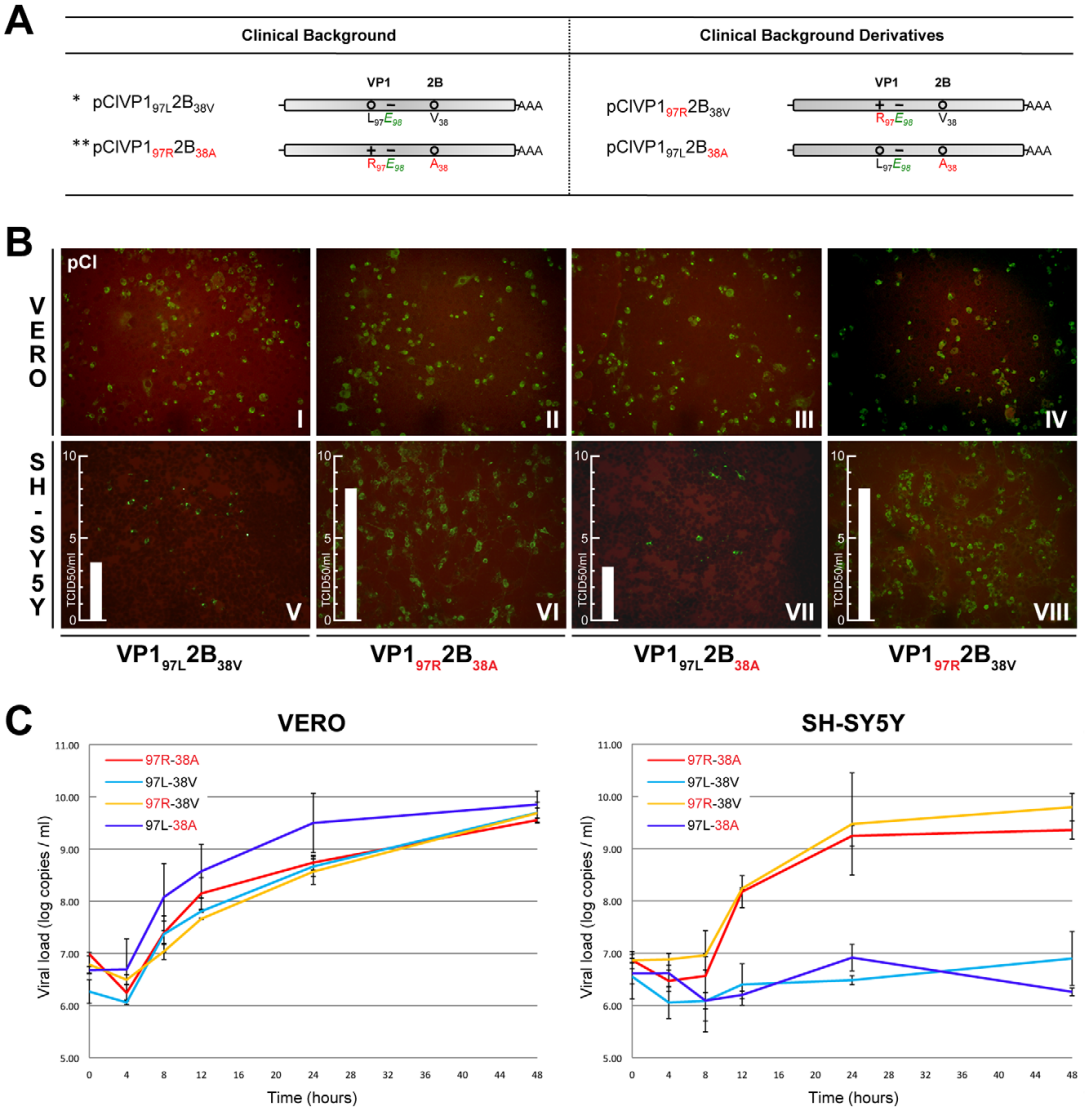
Schematic representation of the infectious clones derived from the stool and BAL isolates and their derivatives and assessment of their replication efficiency in Vero versus SH-SY5Y cells. (A) Representation of the infectious clones. (B–C) Vero cells (I–IV) and SH-SY5Y cells (V–VIII) were infected with infectious clones derived from the clinical isolates. (B) Replication was assessed by immunofluorescence with an anti-EV71 monoclonal antibody. The cell type and residue at positions VP1_97_ and 2B_38_ are indicated on the left and bottom of the figure, respectively. The TCID50/ml performed after 5 days on the SH-SY5Y infected cell supernatant is indicated with a bar on the left side of each panel. (C) Increase in viral RNA load as quantified by real-time RT-PCR at different time points post infection with the four derivatives. Vertical bars indicate minimum/maximum values. Key for (A) +, positive charge, − negative charge, o neutral; * sequence corresponding to that of the BAL samples, ** sequence corresponding to that of the stool samples.

To assess the implication of each of the two non-synonymous substitutions governing the replicative advantage of the stool isolate in cells of neuroblastoma origin, we designed four infectious clones strictly similar to the full-length sequences of the stool or the BAL specimens, as well as two that harbor either the VP1_97R_ or the 2B_38A_ substitutions alone (Figure 2A). We analyzed the replication efficiency of these four constructs in neuroblastoma cells (Figure 2B–2C) and other cell types (Table 3). As expected, and in contrast to the VP1_97L_2B_38V_ and VP1_97L_2B_38A_ clones that presented a delayed growth phenotype in SH-SY5Y, the virus with the stool isolate sequence and the clone with the VP1_97R_ substitution only grew very efficiently in Vero and SH-SY5Y cells, suggesting that the VP1 L97R substitution confers a replicative advantage in neuroblastoma cell line. The immunofluorescence results were corroborated by single-step replication analysis of the four derivatives in Vero and SH-SY5Y cell lines. Although the four derivatives demonstrated similar growth kinetics in Vero cells, the derivatives with the VP1L97R substitution (pClVP1_97R_2B_38A_ and pClVP1_97R_2B_38V_) presented a strong replicative advantage in SH-SY5Y cells (Figure 2C). A significant difference linked to the VP1 sequence was also observed in cells of colorectal adenocarcinoma origin (Caco-2) with the opposite phenotype in this cell line since the growth advantage was conferred by the VP1_97L_ sequence (Table 3).

**Table 3:** Cell tropism of the different clinical isolates infectious clone derivatives.

Origin of viral stock	Cell lines				
	Vero	H292	Caco-2	SH-SY5Y	U-87 MG
	Monkey kidney	Lung carcinoma	Colorectal adenocarcinoma	Neuroblastoma	Astrocytoma, glioblastoma
*pClVP1_97L_2B_38V_	14.91 (±2.17)	2.17 (±0.01)	59.52 (±0.29)	2.6 (±1.97)	7.25 (±4.8)
**pClVP1_97R_2B_38A_	12.10 (±1.22)	7.7 (±3.98)	4.8 (±0.93)	40.65 (±12.41)	4.55 (±1.1)
pClVP1_97L_2B_38A_	10.51 (±0.49)	5.51 (±4.47)	43.96 (±8.98)	0.8 (±0.2)	5.87 (±0.18)
pClVP1_97R_2B_38V_	14.91 (±0.34)	14 (±2.61)	17.6 (±0.27)	31.01 (±4.1)	7,83 (±3.43)

The percent of infected cells measured by metamorph analysis is indicated with the standard deviation calculated out of two biological replicates (in parenthesis).

*: derives from the bronchoalveolar sequence,

**: derives from the stool sequence.

To further confirm the contribution of the VP1 L97R substitution towards the replicative advantage in cells of neuroblastoma origin, these changes were introduced individually or together in the EV71BrCr (GenBank accession number: AB204852.1) infectious clone background (kindly provided by Prof. M Arita, National Institute of Infectious Diseases, Tokyo, Japan) that presents 81% nt and 96% aa identity with the sequence of the clinical isolates. Of note, a Lys at position 98 that reduced the replication of pBrCr in Vero cells due to the introduction of a positively charged aa was substituted with a Glu to restore normal replication. Although the replication of BrCr derivatives is very low in neuroblastoma cells, the trend was similar to that observed with the four infectious clones (data not shown).

### Competition Experiments

**Figure 3:**
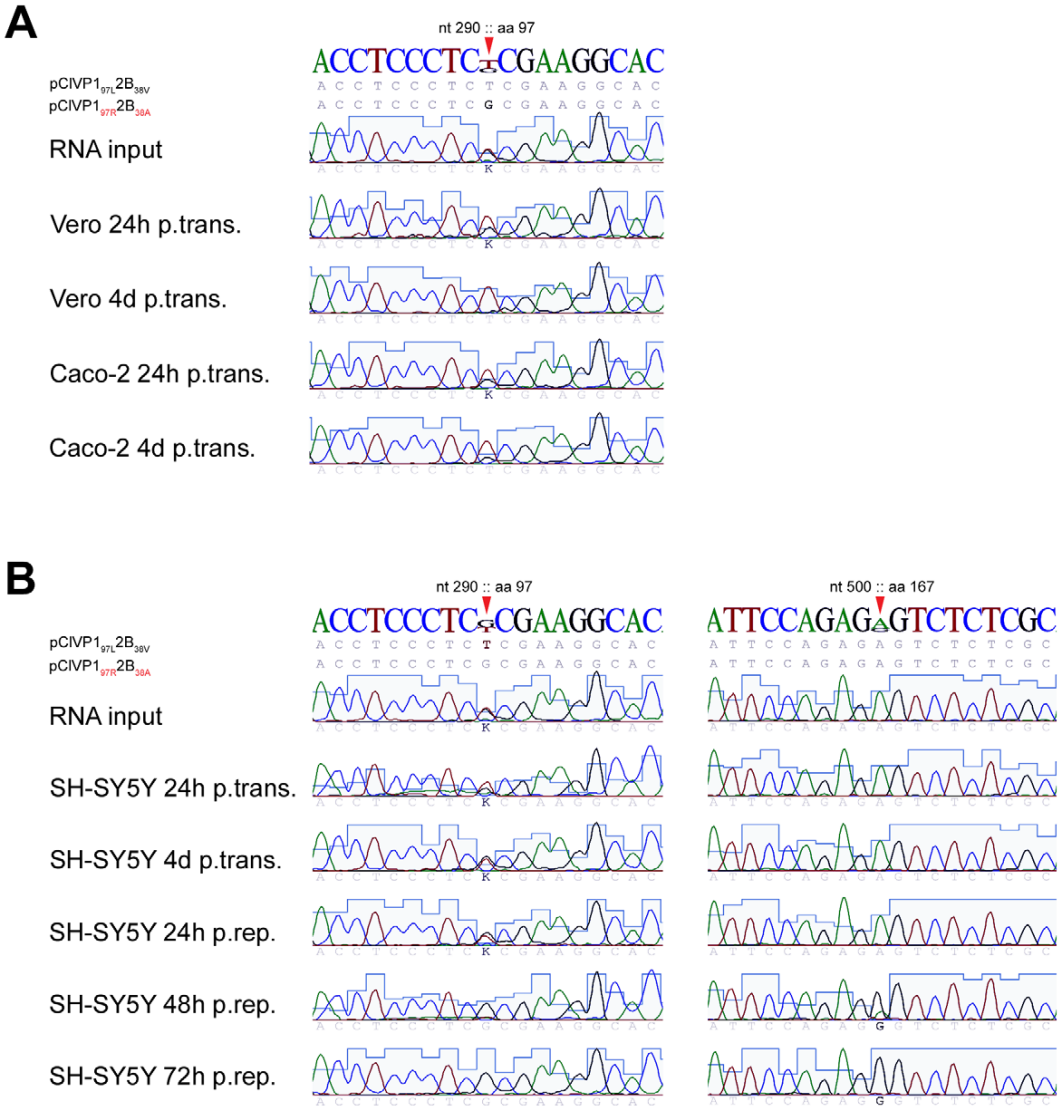
Competition between the stool and BAL infectious clone derivatives. Vero cells, Caco-2 cells (A), and SH-SY5Y cells (B) were transfected by equimolar amounts of the stool and BAL infectious clone derivatives and virus present in the cell supernatant was analysed by sequencing post-transfection and repassage at different times. Substitutions are marked by red arrows and correspond to nucleotide (nt) and amino acid (aa) positions of the EV71 VP1 coding sequence (GenBank accession number: AAB39968.1).

Competition experiments were performed in Vero, SH-SY5Y, and Caco-2 cells to confirm these observations. For this purpose, equimolar amounts of RNA from the infectious clones derived from the stool (pClVP1_97R_2B_38A_) and BAL (pClVP1_97L_2B_38V_) isolates were co-transfected in these three cell lines. The supernatant was collected and viral sequences analysed at different time points post-transfection. As early as 24 h post-transfection, the observed dominant species in Vero and Caco-2 cells was that with VP1_97L_. Regarding SH-SY5Y, at the beginning of the competition (24 h post-transfection) the population with VP1_97L_ appeared to slightly dominate over the VP1_97R_ population. However, the situation reversed after 4 days and the VP1_97R_ population became the dominant species (between 24 and 48 h after the first passage) (Figure 3B, left panel). The fact that the VP1_97L_ sequence dominates shortly after transfection suggests that once inside the cell, this position confers a replicative advantage over the VP1_97R_ sequence. Therefore, the VP1_97R_ sequence likely presents an advantage at the cell entry stage of the viral growth cycle. Interestingly, in two of four competition experiments, the VP1_97R_ substitution was rapidly associated with a second substitution (glutamate to glycine) present at position 167 (E167G) of VP1 (VP1_167G_) (Figure 3B, right panel). By retrospective sequence analysis of SH-SY5Y cells infected with the stool isolate or transfected with the stool or pClVP1_97R_2B_38A_ derivatives, position 167 was almost always mutated into a glycine or an alanine. Alignment of the 952 VP1 sequences currently available in Genbank shows that only one other EV71 sequence (GenBank accession number: AAF13503.1) contains an alanine at position 167. Interestingly this strain also has an arginine at position 97 of VP1 (VP1_97R_).

Finally, to investigate any potential implication of the L97R substitution regarding sensitivity to interferon beta, we co-transfected pClVP1_97R_2B_38A_ and pClVP1_97L_2B_38V_ in Vero cells (that do not produce, but are sensitive to interferon [37], [38]) pre-treated with interferon beta. The viral replication was strongly reduced by the presence of interferon beta and the VP1_97R_ substitution did not provide any advantage to the virus since the pCl VP1_97L_2B_38V_ construct was dominant after 24 h in Vero cells in the presence or absence of interferon (data not shown).

### VP1 Structural Modelling and Virus Binding Assay

**Figure 4:**
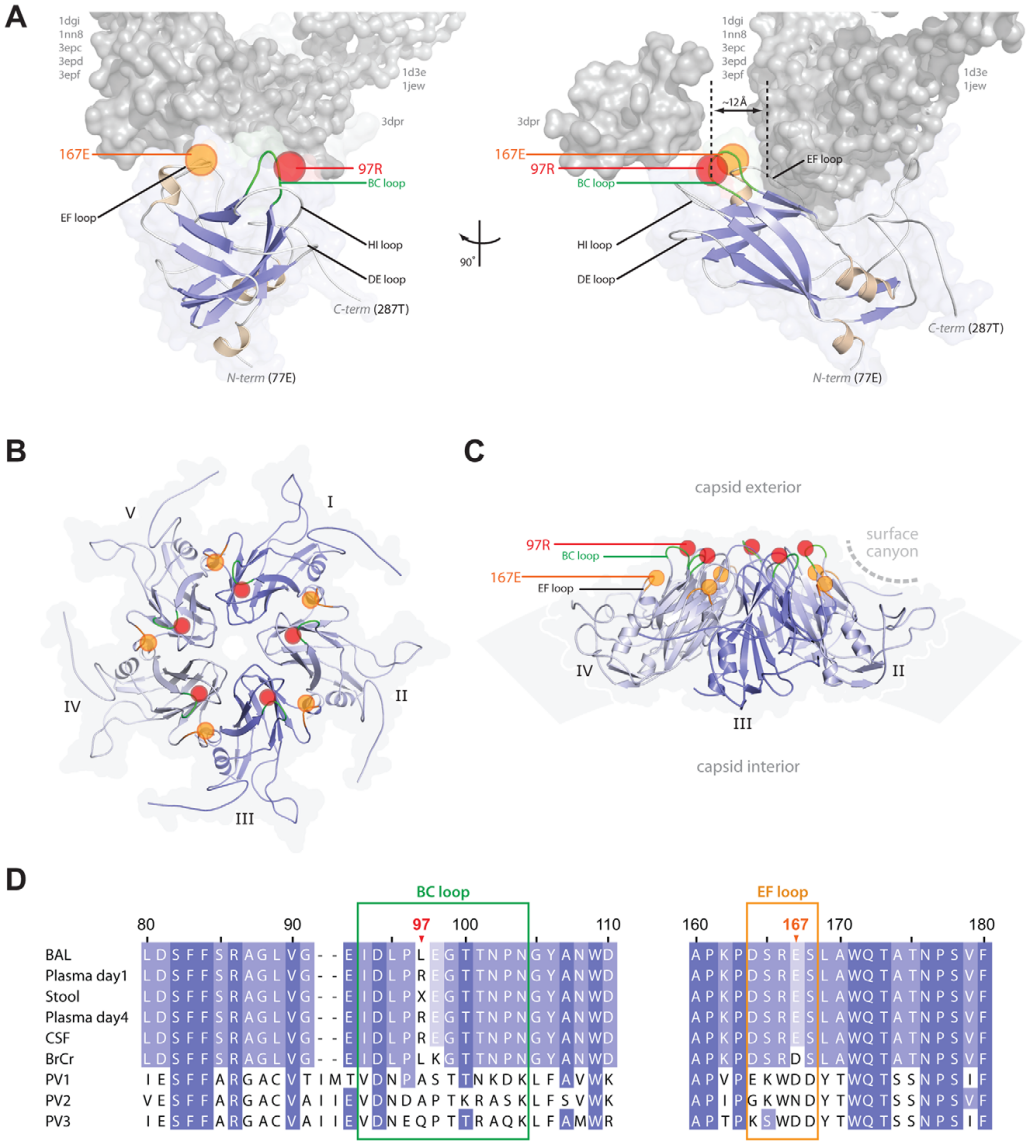
EV71 VP1 substitution locations relative to known receptors and capsid symmetry. (A) EV71 VP1 model highlighting the BC loop (green) and positions of VP1_97R_ (red circle) and VP1_167E_ (orange circle) relative to known receptors (gray). Eight known picornavirus VP1-receptor complexes (PDB codes along their sides) were structurally aligned to our model using the VP1 coordinates in each structure file. The distance (∼12 Å) between EV71 VP1 residue 97 and receptor surfaces is marked by vertical black dotted lines (distance between VP1 residue 97 and the 3dpr receptor, also ∼12 Å, is not marked). (B) Five EV71 VP1_97R_ model monomers arranged in capsid symmetry based on poliovirus capsid VP1 orientations (PDB 3epf). BC loops (green) and positions of residue 97 (red circles) and residue 167 (orange circles) are highlighted. (C) Side view of VP1_97R_ capsid assembly in B, rotated 80° on the plane of this page. The curvature and thickness of the capsid surface (based on PDB 3epf capsid assembly, VIPERdb) is represented as a light gray arc. (D) Sequence alignment of VP1 clinical isolates, EV71 substrain BrCr (Genbank U22521), and polivirus (PV1 (Genbank V01149), PV2 (M12197), PV3 (K01392)) surrounding EV71 VP1_97_ and VP1_167_ substitutions. Index numbers refer to EV71 VP1 residue positions.

**Figure 5:**
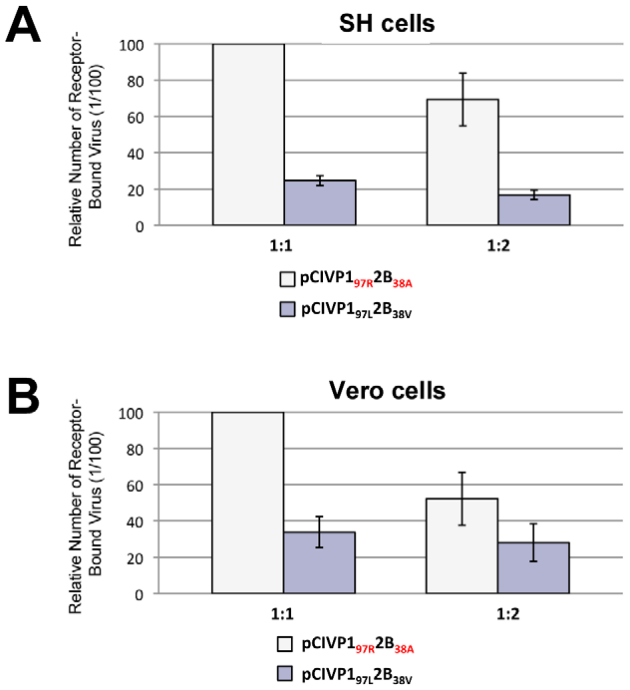
EV71 binding assay. SH-SY5Y cells (A) and Vero cells (B) were used to compare the cell-binding capacity of pCIVP1_97R_2B_38A_ and pCIVP1_97L_2B_38V_. Two conditions were assessed: undiluted (1∶1) and 2-fold diluted (1∶2) standardized viral stocks. Quantification of bound virus was measured by the Entero/Ge/08 real-time RT-PCR assay and expressed relative to pCIVP1_97R_2B_38A_ (1∶1 condition). Vertical bars indicate minimum/maximum values.

To determine if the substitution at VP1 residue 97 could have a structural impact and/or influence how the viral capsid interacts with cellular receptors or co-receptors, we generated and validated comparative models of EV71 VP1_97L_ and VP1_97R_ based on the known VP1 structures of 10 other closely related picornaviruses. Comparison of the energy signatures and structures of the models revealed that VP1_97R_ has no significant energetic or backbone conformational differences relative to VP1_97L_ (data not shown), suggesting that this substitution functions by influencing interactions at the capsid-host cell interface. To further assess this possibility, we aligned all known picornavirus VP1 structures that are in complex with their corresponding cellular receptors. In seven of the eight VP1-receptor structures (PDB accession codes listed in Figure 4A), the receptors bind in a canyon that contains the base of the BC loop, albeit in different orientations. One of the eight structures (PDB 3dpr: a human rhinovirus 2 [HRV2] bound to its receptor) revealed that the receptor interacts with VP1 not in the canyon, but directly above the BC loop at the five-fold axis of symmetry of VP1 (Figures 4A and C). We then aligned our EV71 VP1 models to the VP1 molecules in these structures and observed that EV71 VP1_97_ is within 10–12 Å of the receptor surfaces. Given that amino acid sequences within the BC loop contribute to receptor selectivity among picornaviruses [32], [33] (Figure 4D), and that different strains interact with their receptor in different orientations and regions, we speculated that the positive charge introduced by the VP1_97R_ substitution could be located at the interface of human EV71 receptors and facilitate interactions with host cell receptors. Indeed, after aligning our EV71 VP1 models to the poliovirus VP1 monomers (PDB 3epf) of a complete viral capsid assembly, the arrangement of the VP1 5-mer revealed that residue 97 was close to the five-fold axis of symmetry (Figures 4B and C) in the region known to interact with host cells [39]. This model is further supported by a virus binding assay performed in Vero and SH-SY5Y cells (Figure 5). A difference in binding competence is observed in favour of pCIVP1_97R_2B_38A_ compared to pCIVP1_97L_2B_38V_, which supports the importance of the VP1_97R_ substitution in the receptor–binding process. Of note the VP1_167_ position, where the compensatory E167G mutation occurred *in vitro* in neuroblastoma cells, lies near the interface of VP1 monomers in the capsid assembly (Figures 4B and C). Residue 167 is positioned against another negatively charged residue on the adjacent VP1 monomer and replacement of this glutamate 167 with a glycine might serve to stabilize the capsid assembly by alleviating steric and/or electrostatic interference between VP1 monomers or receptors when position 97 is mutated to an arginine.

## Discussion

Many investigations have focused on the molecular epidemiology of EV71 [19], [20], [31], [40]–[56], but few have attempted to identify in-host adaptation and the potential viral determinant of neurotropism or neurovirulence. Mutations in EV71 5′ UTR [23], [53], [57], VP1 gene (including the BC loop) [31], [53], and 3D polymerase [20], [22] have been shown to result in attenuation in cynomologus monkeys and in mice, but they do not change the tissue specificity in the CNS of these experimental animal models [20]–[22]. These models have many intrinsic limitations, namely, the use of adapted EV71 mouse strains and/or direct intracranial, intramuscular, or intraperitoneal inoculation. These experimental models are thus unable to mimic the natural route of infection in humans.

In this study, we analyzed the genomic differences in the EV71 genogroup C1 virus during a disseminated human disease that included meningitis. EV71 serotypes are divided into three major genetic lineages; lineage A whose prototype is the BrCr strain, and lineages B and C [31] that are further subdivided into subgenogroups B1 to B5 and C1 to C5 [8], [31], [40], [41], [43]. Studies have suggested that the C1 genogroup is rarely a cause of CNS infection [11], [41]. Our goal was to identify viral signatures that could account for dissemination or site-specific adaptation. The comparison of five full-length genomes, sequenced directly from respiratory, gastrointestinal, CSF and blood specimens, revealed a drastic non-synonymous L97R substitution in the BC loop of the VP1 capsid protein that significantly modified the resulting viral phenotype. This mutation was specifically present in the blood and CSF, but not in the respiratory tract, and was present as a mixed population in the gastrointestinal tract. In addition to the VP1_97R_ substitution, a conservative amino acid substitution at position 38 of protein 2B (V38A) was also observed in the blood, CSF, and gastrointestinal tract. Finally, a mixture of two nucleotides, both translated into a leucine in the 3D gene (3D_175_), was also observed in the sequence of the stool specimen.

The BC loop region of VP1 is a known dominant immunogenic site as evidenced from experimental models using laboratory-adapted poliovirus or coxsackie virus strains [24]–[29]. Seroneutralization experiments with the patient's serum failed to highlight the presence of an antibody-mediated, selective immune pressure promoting the VP1 L97R substitution. Thus, it is unlikely that the VP1_97R_ sequence present in the immunocompromised patient's blood and CSF played a critical role in viral dissemination to the nervous system via immune escape. This has to be related to the immunosuppressed condition of the patient who was previously treated with anti-CD20 antibodies, although we cannot rule out the presence of low level antibodies or cellular immunity directed against the VP1_97L_ strain that have gone undetected.

Apart from its immunogenic role, the BC loop region of VP1 was also identified as a determinant of poliovirus host adaptation [32], [33], [58]. *In vitro* cell tropism assays revealed that the VP1_97R,_ conferred a significant advantage to the ability of EV71 to grow in neural-derived cells, independent of the virus lineage. The results of competition experiments suggest that the advantage is probably at the cell entry step. Indeed, introduction of a positively charged amino acid in the BC loop may have a substantial impact on the interaction of host cell surface receptors with this epitope. The EV71 VP1 structure models and the virus binding assay further support this hypothesis by revealing that residue 97 is close (∼12 Å) to the interface of other known picornavirus VP1 receptors (Figure 4A), and by illustrating how the positively charged arginine side chain of VP1_97R_ on the viral capsid surface may be more accessible to certain host cell receptors than the smaller side-chain of leucine of VP1_97L_ (Figure 4C). Of note, backbone carbon atom alignment of our models to the EV71 VP1 structures [59], [60] that were released while this manuscript was under review shows less than 1.0 angstrom RMSD between models and structures (data not shown), thus further validating our modelling approach.

Interestingly, although the improved receptor-binding capacity of VP1_97R_ might be sufficient to confer a growth advantage in neuroblastoma cells, it cannot be ruled out that this substitution also confers potential advantages at various other stages (e.g., during the virion assembly process). This binding advantage was also observed to a lesser extent in Vero cells. Therefore, compensatory events must occur at one or multiple steps during virus amplification (viral genome replication, assembly, recruitment of cellular factors, others) to explain the VP1_97L_ advantage observed in the competition assay in this cell line. Of note, inoculation of the patient's stool specimen (that presented both VP1_97R_ and VP1_97L_ populations) in Vero cells resulted in virus isolate containing only the VP1_97R_ sequence. While this may seem surprising in light of the results from the competition experiment, one must note that Vero cells were transfected with RNA transcripts from pCI derivatives, thus bypassing the viral entry step. According to the binding assay, VP1_97R_ also provides a binding advantage in Vero cells that may partially explain these contradictory observations. Another possible explanation is that while the presence of a mixed population in stools was shown at the RNA level, there is no indication about the viability of the corresponding viral species in the sample. Thus, the presence of a leftover, potentially defective, viral VP1_97L_ genome that is unable to grow in culture cannot be ruled out.

Notably, in neuroblastoma cells, VP1_97R_ was frequently associated with a second mutation located in the EF loop at position 167 (E167G) of VP1 (VP1_167G_). In our structure models, position 167 is situated in the receptor-binding canyon near the base of the BC loop and may serve to limit the conformational flexibility of the BC loop (Figures 4A–C). Substitution of a negatively charged glutamate by the smaller neutral glycine may alleviate steric and/or electrostatic interference created by the VP1_97R_ at the VP1-receptor interface, thus serving to stabilize the VP1 interaction with the host cell receptor. This E167G substitution was absent in all of the patient's specimens analyzed, suggesting that this position is either not clinically relevant and only reflects cell-type adaptation under our experimental conditions, or that there was not enough time for it to appear during the course of infection. If more time had elapsed before treatment was administered, it is possible that the VP1_167G_ substitution would have appeared and, in turn, exacerbated the patient's symptoms. This second hypothesis is favored by the finding of a publicly available sequence that contains both VP1_97R_ and VP1_167G_ residues (GenBank accession number: AAF13503.1).

*In vivo* studies in mouse models [61]–[63] and a comparative analysis of all EV71 complete genome sequences with identified clinical backgrounds available in the Genbank database [64] both identified amino acid positions in VP1 associated with EV71 virulence, such as VP1_145_ in the DE loop situated on the rim of the surface canyon, or VP1_164_ in the EF loop situated on the slope of the canyon (Figure 4). Furthermore, after amplification of infectious clones harboring EV71 subgenogroup B3 in SH-SY5Y and RD cell lines, VP1_94_ was recently identified and is postulated to be important for cell-type adaptation [36]. Taken together, the region surrounding the VP1 L97R mutation identified in this study likely plays an important role in cell-type adaptation and potentially neurotropism, independent of the EV71 genogroup.

In addition to the VP1_97R_ substitution, a conservative amino acid substitution at position 38 of protein 2B was observed, 2B_38A_. This substitution is uncommon and described in only one case among all available GenBank sequences. Whether this conservative V38A change in protein 2B may also confer new viral tropism was not substantiated in our experiments and remains an unsupported hypothesis.

Taken together, the sequence of clinical events, the genome characterization, our *in vitro* experiments, and our comparative VP1 structure models support the following scenario: the virus could have initially infected the respiratory tract, leading to a first viremic phase followed by invasion of the gastrointestinal tract. Alternatively, the virus may have entered simultaneously by oro-fecal and respiratory routes. High replication in the gastrointestinal tract may then have given rise to the appearance of a mixed viral population. The reduced immune response of the host then allowed a prolonged viremia, originating from a subspecies generated during the replication within the gastrointestinal tract that conferred a selective advantage for certain cell types, including neural cells. This resulted finally in neuro-invasion. In conclusion, this study provides the first genome-wide analysis of EV71 evolution and dissemination within a single human host over the course of an infection, and highlights how emergence of mutations at critical regions of the viral genome can lead to new phenotypes and neurovirulence. Further studies are underway to better define the target of the VP1_97R_ substitution and to investigate any potential effects of the associated mutation, VP1_167G_.

## Materials and Methods

### Ethics Statement

The study was approved by the institutional ethics committee of the University Hospitals of Geneva, Switzerland. Given the nature of the investigation, that none of the sampling was done for the purpose of this investigation, and that enterovirus genotyping is part of our routine surveillance activity, the requirement of written consent was waived by the ethical review board. Oral informed consent was obtained from the patient concerning the fact that the infective virus would be characterized.

### Human Specimens

The following specimens were collected for diagnostic purposes at different times in a patient hospitalized with a disseminated EV71 infection: a bronchoalveolar lavage fluid (BAL) was collected upon patient admission; a blood and stool specimen were collected 17 h and 19 h later, respectively, as well as a second blood sample after 4 days; and a cerebrospinal fluid (CSF) sample after 5 days.

### RNA Extraction, Reverse Transcription and Real-Time Polymerase Chain Reaction (PCR)

RNA extraction in blood was performed with the NucliSens miniMAG method, according to the manufacturer's instructions (bioMérieux, Geneva, Switzerland). RNA extraction in BAL, CSF, and feces was performed with TRIzol (Invitrogen, Carlsbad, CA, USA). RNA extraction from infected cell and infected cell supernatant was performed with easyMAG (bioMérieux). Reverse transcription was carried out with the Superscript II RNase H^−^ enzyme (Invitrogen) with both random hexamers and oligodT (for the most 3′ part of the genome). Real-time RT-PCR enteroviral screening was then performed with Taqman Universal Mastermix (Applied Biosystems, Rotkreuz, Switzerland) with primers and probe sequences described previously [65] and Entero/Ge/08 [66].

Amplification and detection were achieved with ABI Prism 7900 and 7000 sequence detection system (Applied Biosystems) according to methods previously described [67].

For single-step replication quantification, the Entero/Ge/08 assay was used in a one-step format using the QuantiTect Probe RT-PCR Kit (Qiagen, Hombrechtikon, Switzerland) according to the manufacturer's instructions in a 7000 Applied Biosystems thermocycler. For each derivative, viral amplicon Ct values were normalized according to the input RNA amount present in the inoculum and then to those of the endogenous RNase P gene (TaqMan RNase P Control Reagents, Applied Biosystems). Relative quantification was calculated using the 2^−ΔΔCt^ method [68]. The quantitative Entero/Ge/08 assay was run using a 10-fold dilution series (from 5*10^7^ to 5*10^4^ copies/ml) of the *in vitro* transcribed full-length pClVP1_97R_2B_38V_ derivative, which was used as a quantitative reference curve for each run.

### PCR and Sequencing

Overlapping fragments representing the complete viral genome were amplified by PCR using the AmpliTaq polymerase (Applied Biosystems) and primers designed on the basis of previously published EV71 strains. Specific primers were then designed to fill the gaps. All primers used are listed in Table S1. PCR products were purified and sequenced as previously described [67]. Each product was sequenced at least twice and analyzed by vector NTI Advance 10 software (Invitrogen). Ambiguous nucleotides were resolved by re-sequencing. To avoid the introduction of mutations by cell culture adaptation, full-length sequences were obtained directly from the clinical samples. The three nucleotide differences observed between the samples were confirmed by a new cycle of PCR and sequencing.

### Virus Isolation, Titration and Seroneutralization Assays

Diluted stool and BAL specimens were used to inoculate Vero-76 cells in 1.6 ml infection medium (Dulbecco plus 2.5% fetal calf serum, 0.2% sodium bicarbonate, penicillin, streptomycin, fungisone, gentamicin, and Hepes). Viral stocks were prepared after three cell passages. To confirm the conservation of the substitutions, partial VP1 and 2B amplifications and sequencing of the stocks were carried out with primers AN89 and AN88 [69] and primers 30 and 15, respectively, and sequenced with the PCR primers (Table S1). The stocks were quantified at day 7 post infection according to the Reed and Muench method [70] and presented a 10^5.7^ and 10^5.62^ TCID50/ml for the stool and lower respiratory samples, respectively.

For seroneutralization assays, 100 uL of virus diluted stocks (dilution factor 10^4^) were incubated with 100 uL of diluted patient serum sampled at day 4 or with 100 uL of mouse anti-EV71 monoclonal antibody (MAB979; Chemicon, Temecula, CA, USA); antibody dilutions were 1/5, 1/10, 1/15, 1/20 and 1/30. The mix was incubated for 1 h at 37°C before inoculation on Vero cells and then incubated at 37°C for one week. Each dilution was performed in duplicate and the experiment was repeated twice. Control dilutions of the virus in the absence of antibody were performed in the same experiment. The cytopathic effect was read daily. The inhibition titre was defined at day 8 post inoculation. The BC loop of the 2 viral isolates was sequenced after neutralization with the patient serum to exclude reversion mutation.

### Construction of pEV71 Infectious Clones

Four infectious clones containing the stool sequence (pClVP1_97R_2B_38A_), the lower respiratory tract sequence (pClVP1_97L_2B_38V_), and two hybrid sequences (pClVP1_97R_2B_38V_ and pClVP1_97L_2B_38A_) cloned with MluI/BamH1 in a modified pcDNA3.1 vector were ordered at Biomatik (Ontario, Canada).

### Preparation of Quantified Stocks from Infectious Clones and Competition Experiments

*In vitro* transcription of pCl plasmids linearized with *BamH1* were performed as previously described [71]. Vero cells were seeded at 6×10^5^ cells in 35 mm wells of a 6-well plate. The following day, cells were transfected with 2 ug of RNA transcripts containing the different pCl derivatives using the TransMessenger Transfection Reagent kit (Qiagen). After 3 h at 37°C, the infection medium (see below) was used to replace the transfection mix. Cells were then incubated at 37°C. For pCl derivatives, cell supernatants were collected when the cytopathic effect was noticeable and passaged once in 24 cm^2^ cell culture flasks. Cells were incubated at 37°C until appearance of cytopathic effects (3–4 days post-transfection). Supernatants were then immediately clarified 5 min at 1000 rpm in a Multifuge 4 KR, aliquoted and stored at −70°C. The stocks were quantified according to the Reed and Muench method [70].

For competition experiments, equimolar amounts of RNA from pClVP1_97L_2B_38V_ and pClVP1_97R_2B_38A_ were co-transfected in Vero cells pretreated with 8 U/ml of Interferon beta (Rebif; Merck Serono, Geneva, Switzerland) for 12 h before transfection, SH-SY5Y cells, and Caco-2 cells. Cell supernatants were then collected at different time points and repassaged. The input RNA and the RNA extracted from cell supernatants collected at different time points were reverse transcribed and analyzed by PCR and sequencing.

### Cell Tropism Assay

Vero-76 (ATCC # CRL-1587), H292 (ATCC # CRL1848), Caco-2 cells (ATCC # HTB 37), SH-SY5Y (ATCC #CRL-2266), SK-N-MC (ATCC #HTB10), and U-87 MG (ATCC #HTB14) cell lines were used for the cell tropism assay. The infection medium for the HEp-2 cells is the same as the infection medium for Vero cells (see above). For SK-N-MC and U-87 MG cells, Dulbecco is replaced with McCoy medium for SH-SY5Y with RPMI 1640, with M100 supplemented with 10% fetal calf serum (FCS) for Caco-2 cells, and Eagle's minimum essential medium (EMEM) supplemented with 10% FCS for H292.

For pCl derivatives, replication was assessed by immunofluorescence 36 h post-infection for SH-SY5Y and after 48 h for Vero, H292, Caco-2, SK-N-MC, and U-87 MG cells. Virus isolated from SH-SY5Y and Vero cell supernatants were analysed by complete genome sequencing and quantified by the Reed and Muench method [70]. Replication in Vero and SH-SY5Y cells was further analyzed by real-time RT-PCR on RNA extracted from total cell lysates 4 h, 8 h, 12 h, 24 h and 48 h post-infection. Infections were performed in duplicate for each time point.

### Immunofluorescence

EV71-infected cells were labelled as follows: cells were washed twice with phosphate buffered saline (PBS) lacking Ca^2+^ and Mg^2+^ (PBS^−^) and fixed 1.5 h in methanol-acetone (50∶50) at −20°C. Cells were air-dried for a few minutes at room temperature before incubation with the mouse anti-EV71 (MAB979; Chemicon) primary antibody diluted 1/40 in PBS^−^-1% bovine serum albumin (BSA), for 45 min at 37°C in a humidity chamber. After intensive washing with PBS^−^, the anti-mouse IgG AB/FITC containing 0.02% Evans Blue counterstain (Millipore-Light Diagnostics, Zug, Switzerland) was added and the cells were incubated for 45 min at 37°C in the dark. After final rinsing with PBS^−^, coverslips were mounted in fluorotec embedding medium (BioScience AG, Rüschlikon/Zurich, Switzerland). Quantification of virus growth was calculated either manually as the percentage of positive cells or by metamorph analysis.

### Metamorph Analysis

Images were acquired on a Zeiss AxioCam microscope with 20× and 10× objectives, leading to the calibration of 0.33 µm/pixel and 0.67 µm/pixel. Images were acquired in 2400×2500 spatial resolution and 24 bit color depth (8 bit/channel). To measure the positive markers, the following image analysis was performed with Metamorph/MetaXpress software (Molecular Devices, Sunnyvale, CA). The blue channel of the images contained DAPI-stained nuclei and the positive cells were marked with antibody-GFP. The first step of processing involved separating the two channels (blue and green); respective channels were converted from 8 to 16 bit by multiplication and the “CellScoring” tool of Metamorph software was applied to 16 bit versions of blue and green channels. Parameters used for images at 10× magnification were as follows: cell minimum width, 7; cell maximum width, 20; intensity above local threshold, 20. For images taken with the 20× objective, the respective parameters were: cell minimum width, 13; cell maximum width, 40; intensity above local threshold, 20. For both series, the positive marking was sought in the cytoplasm (parameter “Stained area”). Reported parameters include: total cell number; positive cell number; and their relative percentage.

### Virus Binding Assay

SH-SY5Y and Vero cells were seeded at 2×10^4^ and 4×10^4^ cells/well, respectively, in 96-well plates. The following day, culture medium is removed and cells are washed once with cold Hanks' Balanced Salt Solution (HBSS) with CaCl2 and MgCl2 (Invitrogen). 200 µL of binding buffer (HBSS containing 1% BSA and 0.1% sodium azide) are then added and cells chilled on ice for 10 min. Supernatant is removed from cells. 100 µL of pCIVP1_97R_2B_38A_ and pCIVP1_97L_2B_38V_ stocks (amplified in Vero cells) standardized by the Entero/Ge/08 real-time RT-PCR (further confirmed by sequencing-chromatogram ratios analysis of standardized pCIVP1_97R_2B_38A_and pCIVP1_97L_2B_38V_ pooled stocks) are added. After 1 h of incubation on ice, unbound virus is removed by three wash steps with 200 µL/well of cold binding buffer and then cells are lysed in the wells with 200 uL of easyMAG lysis buffer. Viral RNA is extracted with the NucliSens miniMAG method and detected by real-time RT-PCR using the Entero/Ge/08 assay. RNase P quantification by Taqman assay (Applied Biosystems) was used for normalization. The virus binding assays were performed systematically in duplicate in two individual experiments for each condition.

### Comparative Protein Structure Modelling

The EV71 VP1 models were generated and evaluated using the molecular modelling suite MODELLER v9.9 [72]. As input for the modelling algorithm, our clinically isolated EV71 VP1 amino acid sequences were used to find homologous viral VP1 structures in the Protein Data Bank [PDB]. Five structures with the highest percentage identity to the EV71 VP1 amino acid sequence were selected to serve as initial model templates; bovine enterovirus VG-5-27 (PDB accession code: 1bev, 46% identity to EV71 VP1), coxsackievirus B3 coat protein (1cov, 42%), swine vesicular disease virus (1fpn, 42%), human rhinovirus serotype 2 (1r1a, 43%), and serotype 1A (1oop, 42%). The EV71 VP1 models were evaluated using the GA341 potential and the discrete optimized protein energy (DOPE) algorithms of MODELLER v9.9 [73]. While a high degree of structural variability was found in the N- and C- termini of the EV71 VP1 models, the ‘core’ VP1 sequence that is exposed and interacts with the host environment is highly similar in all template structures (core EV71 VP1 models have an overall root mean square deviation (RMSD) of ∼0.25 Å for backbone carbon atoms). In a second round of modelling, the core EV71 VP1 sequence (residues 77 to 287) was used to select 10 homologous template structures from the PDB that yielded a set containing the original five structures with human rhinovirus 16 (1aym, 42% identical to EV71 VP1 core residues), poliovirus type 2 (1eah, 42%), Sabin strain poliovirus PV3 (1pvc, 41%), swine vesicular disease virus (1mqt, 40%), and human coxsackievirus (1z7s, 40%). EV71 VP1 models were generated using these 10 template structures and assessed with GA341 and DOPE algorithms.

To investigate the VP1 models in the context of the five-fold axis of symmetry found in viral capsid assemblies, the EV71 VP1 models were structurally aligned to each VP1 monomer of the poliovirus VP1 (PDB accession code: 3epf) as arranged in the half-capsid structural coordinates found in the Virus Particle Explorer database VIPERdb2 [74] using PyMOL version 1.4.1. (The PyMOL Molecular Graphics System, Version 1.3, Schrödinger, LLC, New York, NY).

## Supporting Information

Table S1 Primers used in this study.
(DOCX)
