# Supplementary material for: Identification of Site-Specific Adaptations Conferring Increased Neural Cell Tropism during Human Enterovirus 71 Infection
Source: PLoS Pathog. 2012 Jul 26;8(7):e1002826. doi: 10.1371/journal.ppat.1002826 (PMC3406088; doi:10.1371/journal.ppat.1002826)
Supplement: Table S1 — Primers used in this study. (DOCX) [file ppat.1002826.s001.docx]

|  | Sequence 5’-3’ | Template sequence | Position* |
| --- | --- | --- | --- |
| 1 | TTAAAACAGCTGTGGGTTGTTCCC | EV71 | 1 |
| 2 | CATAGTTGGCTATGGTGAGTGGCC | EV71 | 1046 |
| 3 | CATCCGGGAATTTCCAGTACCACCC | EV71 | 1207 |
| 4 | AAGTAGTGAAACTGTGCATTCTGGCC | EV71 | 1256 |
| 5 | GTGCCATATATGAACACACTGCCTTTC | EV71 | 1536 |
| 6 | GGTTTTAACTCAGTGGGGAAACCTTG | EV71 | 1739 |
| 7 | GCGACCGCCATGTTGGGTAC | EV71 | 2174 |
| 8 | CATCTGGGATTTTGGGCTACAATC | EV71 | 2180 |
| 9 | TGGTATCAAACAAACTACGTGGTTCC | EV71 | 2304 |
| 10 | TCCCCCTGAATAGAGGCTGTCTG | EV71 | 2452 |
| 11 | TTTGGTCAGCAATCTGGGGC | EV71 | 3339 |
| 12 | CAACGTGCAATTGTGTCACAACC | EV71 | 3500 |
| 13 | GATGCCCCCACAATCACCAG | EV71 | 3672 |
| 14 | GGGAGAAGATTGTACCAGCGGC | EV71 | 4177 |
| 15 | CCAAACATTGCTTCAAGATCTTCTTGT | EV71 | 4313 |
| 16 | GCAGATCCGCTATTGGCAACAC | EV71 | 5026 |
| 17 | ACCTCCTCGCTATCTACACTAGCGAG | EV71 | 5165 |
| 18 | CTACAAAAGCTGGGCAGTGTGG | EV71 | 5812 |
| 19 | AAACACATCATGGAATACACTGGGTTC | EV71 | 6042 |
| 20 | TCCGGTGTGGTTCAGGGCT | EV71 | 6665 |
| 21 | GAATTGAAGATGGAAGTGCCAGAGCACCC | EV71 | 6835 |
| 22 | GCTATTCTGGTTATAACAAATTTACCCCCA | EV71 | 7411 |
| 23 | GGTGCGAAGAGCCTATTGAGCTAGTTGGTAG | Isolate | 418 |
| 24 | GTACCATACATAAACGCACTGCCTTTTG | Isolate | 1536 |
| 25 | TTCAGAGCCGACCCTGG | Isolate | 1971 |
| 26 | CCCTATCTCCCTGAATAGTGCCTGTCTG | Isolate | 2446 |
| 27 | GCACAGCACAGCTGAGACC | Isolate | 2657 |
| 28 | TGCCTTCGCGAGGGAG | Isolate | 2740 |
| 29 | GATCCCCCAGCACAGGTCTCA | Isolate | 2994 |
| 30 | TTCGGACAACAATCCGGGGCC | Isolate | 3339 |
| 31 | CTGGGTTTTGAAAAGCTGACC | Isolate | 3572 |
| 32 | CGCGGCGAGTGCCGC | Isolate | 4109 |
| 33 | GGCGAGTGCCGCTAAAGGG | Isolate | 4112 |
| 34 | GGTCAGATACAGTGTGGACACAGTGGT | Isolate | 4973 |
| 35 | GAGGTTCGCCAATACTGCAG | Isolate | 5160 |
| 36 | CTGCAGTATTGGCGAACCTC | Isolate | 5180 |
| 37 | ATAAACGAGGGACACAACTGCTACCAC | Isolate | 5295 |
| 38 | ATGCTAGGAGTGCGAGATCACCTGG | Isolate | 5469 |
| 39 | AGTCACAACACCACCACACTG | Isolate | 5847 |
| 40 | GACCCAAGGTTGGAGGTTG | Isolate | 6078 |
| 41 | GCTTCCAGGTTCTCTGTACCATAGCA | Isolate | 6266 |
| 42 | GGATGTAACCCAGATGTGTTTTGGAG | Isolate | 6570 |
| 43 | TATGATGCTAGTATCAGCCCAGTGTGG | Isolate | 6648 |
| 44 | TTGAAAATGGAGGTGCCTGAGCAACC | Isolate | 6833 |
| 45 | GTTCAATTCATCGAGATCTATCCC | Isolate | 6909 |
| AN89 | CCAGCACTGACAGCAGYNGARAYNGG | All EV | 2602 |
| AN88 | TACTGGACCACCTGGNGGNAYRWACAT | All EV | 2977 |
|  |  |  |  |
|  |  |  |  |
|  |  |  |  |
|  |  |  |  |
|  |  |  |  |

* Positions relative to EV71
